# Recent Advancements in Ultrasound Transducer: From Material Strategies to Biomedical Applications

**DOI:** 10.34133/2022/9764501

**Published:** 2022-05-11

**Authors:** Jiapu Li, Yuqing Ma, Tao Zhang, K. Kirk Shung, Benpeng Zhu

**Affiliations:** ^1^Wuhan National Laboratory for Optoelectronics, Optics Valley Laboratory, School of Optical and Electronic Information, Huazhong University of Science and Technology, Wuhan, China, 430074; ^2^State Key Laboratory of Transducer Technology, Chinese Academy of Sciences, Shanghai 200050, China; ^3^NIH Resource Center for Medical Ultrasonic Transducer Technology, Department of Biomedical Engineering, University of Southern California, Los Angeles, CA 90089, USA

## Abstract

Ultrasound is extensively studied for biomedical engineering applications. As the core part of the ultrasonic system, the ultrasound transducer plays a significant role. For the purpose of meeting the requirement of precision medicine, the main challenge for the development of ultrasound transducer is to further enhance its performance. In this article, an overview of recent developments in ultrasound transducer technologies that use a variety of material strategies and device designs based on both the piezoelectric and photoacoustic mechanisms is provided. Practical applications are also presented, including ultrasound imaging, ultrasound therapy, particle/cell manipulation, drug delivery, and nerve stimulation. Finally, perspectives and opportunities are also highlighted.

## 1. Introduction

Due to its exclusive advantages, such as safety, low cost, and convenience, medical ultrasound plays an important role in the biomedical engineering field [1]. Ultrasound, when used with different intensities, identifies various physical characteristics. High-intensity ultrasound is able to induce a thermal effect, which is quite useful for tumor ablation [2–5]. With its intensity decreasing, ultrasound successively behaves with mechanical and wave characteristics. Ultrasound radiation force can be utilized for cell/particle manipulation, drug delivery, and neuromodulation [6–9]. Additionally, ultrasound wave, which has the capability to obtain information of a target through echoes, is most commonly used for biomedical imaging [10, 11].

An ultrasound transducer is indispensable for various ultrasonic biomedical applications. The traditional ultrasound device is a type of a piezoelectric transducer that converts electricity into vibrations, thereby generating ultrasound. With the recent development of piezoelectric materials [12–19], a piezoelectric transducer’s performance has been enhanced. Furthermore, some unique technologies, such as 3D printing and stretchable electrodes, provide new insights for ultrasound transducer fabrication [20, 21]. Specifically, ultrasound imaging resolution is directly proportional to its frequency. The higher the frequency, the better the ultrasound resolution. Commonly, clinical ultrasound’s frequency is set in the range of 2-15 MHz, and this can be a problem as this range can result in limited image resolution. To meet the requirement of precision medicine, one research trend for piezoelectric transducers is increasing its operational frequency [22]. Another problem lies in the fact that piezoelectric transducers are easily affected by electromagnetic interference. As a vital supplement to the piezoelectric transducer, the optoacoustic transducer has attracted much attention recently because of its antielectromagnetic interference and easy fabrication process. Such a device converts pulse laser into ultrasound, and its principle is the photoacoustic effect that is discovered by Bell in 1880. Laser-induced ultrasound is also being studied for biomedical imaging, nerve stimulation, cell manipulation, and other biomedical uses [10, 23, 24].

**Figure 1:**
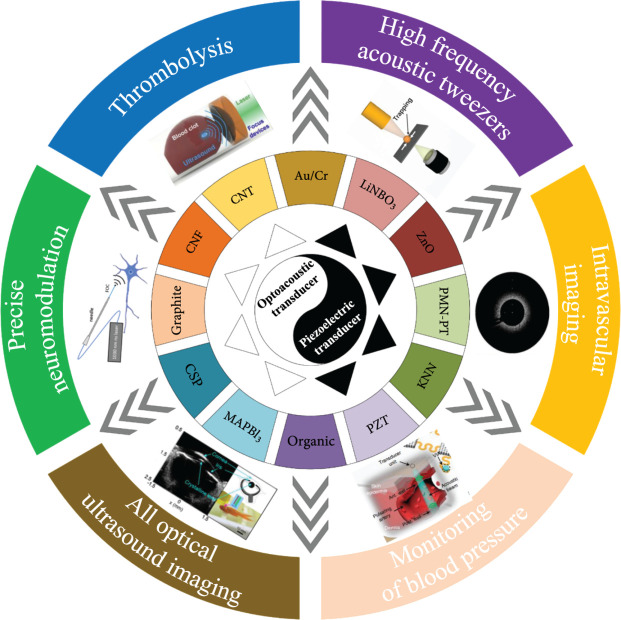
Schematic diagram showing the main topics of piezoelectric/optoacoustic transducers [10, 22, 23, 25–27], reproduced with permission.

This review is aimed at summarizing and classifying recent advances in ultrasound transducer technology that use a variety of material strategies and device designs for biomedical applications. First, ultrasound generation mechanism examples, including electroacoustic and photoacoustic conversion, is discussed. Second, extensive studies on two types of ultrasound transducers from the perspective of material strategies (e.g., piezoelectric ceramics, single crystal, films, composites, and metal-polydimethylsiloxane (PDMS) composite, carbon nanomaterial-PDMS composite, and lead halide perovskite-PDMS composite), fabrication techniques (e.g., 3D printing, dice-and-fill method, and flexible technologies), and biomedical applications (e.g., imaging, cell/particle manipulation, drug delivery, and neuromodulation) are reviewed, as summarized in Figure 1. Finally, opportunities and challenges are presented, and the outlook for future research will be highlighted in the conclusion section.

## 2. Principle of the Ultrasound Generation

### 2.1. Principle of the Piezoelectric Transducer

**Figure 2:**
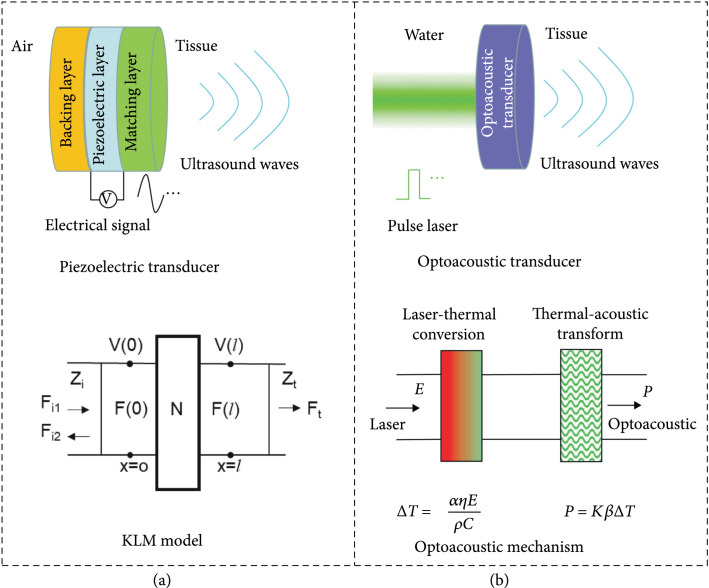
The principle of (a) piezoelectric transducer and (b) optoacoustic transducer.

A piezoelectric transducer belongs to a group of electric-driven devices and often has a three-layer structure (i.e., piezoelectric layer, backing layer, and matching layer), as shown in Figure 2(a). The piezoelectric coefficient ($d_{33}$) and the electromechanical coupling coefficient ($k_{t}$) of the piezoelectric materials have drastic influence on the acoustic performance of a piezoelectric transducer. In particular, the acoustic impedance of piezoelectric materials is ~30 MRayl, which is much higher than that of biological tissues (i.e., ~1.5 MRayl). The acoustic impedance mismatch will cause ultrasound’s reflection at the interface; hence, the ultrasound wave cannot effectively travel through the interface [28, 29]. Therefore, the acoustic matching layer is required, which can improve the sensitivity, bandwidth, and energy transmission efficiency of the transducer. The proposed thickness of the impedance matching layer is $\lambda_{m}/4$ (where $\lambda_{m}$ is the sound wavelength in the matching layer). When the matching layer’s acoustic impedance satisfies Equation (1), the theoretically forward-propagating sound wave can completely pass through the acoustic matching layer [28–30]:

(1)Zm=Zpn−m+1Zcmn+1.

Here, $Z_{\text{p}}$ and *Z*_c_ are the acoustic impedance of the piezoelectric material and coupling medium, respectively. The number of the matching layers is n, and the acoustic impedance of the mth layer is ${\text{Z}}_{m}$. The backing layer (e.g., conductive epoxy) can be used on the rear of the transducer to dampen the echo to reduce the pulse duration and absorb part of the energy from the backward sound wave [28–31]. Usually, an equivalent circuit (KLM) is employed to design piezoelectric transducers [32]. In the KLM model, a piezoelectric transducer is described as a set of finite length transmission lines with a frequency-dependent electroacoustic coupling transformer; here, plane acoustic waves propagate in both directions (Figure 2(a)).

### 2.2. Principle of the Optoacoustic Transducer

The core part of an optoacoustic transducer is optoacoustic material, which usually comprises light absorption material and thermal expansion material (Figure 2(b)). Light absorption material achieves photothermal conversion (ΔT) through a nonradiative transition mechanism. Meanwhile, thermal expansion material emits ultrasonic waves (P) through periodic thermal expansion based on the thermoelastic principle. Thermal conductivity influences the heat transfer between the light absorption and thermal expansion materials and further impacts the optoacoustic energy conversion efficiency and frequency. Before saturation, the output sound pressure is positively correlated with the laser energy density. Furthermore, the pulse laser width determines the upper limit of bandwidth of the optoacoustic signal [33]. The temperature change (ΔT) of an optoacoustic transducer caused by the pulse laser can be expressed as [10, 34, 35]

(2)∆T=αηEρC,

where α, ρ, and C are the absorption coefficient, density, and specific heat capacity of the material, respectively, E is the density of laser energy, and η is the nonradiative transition probability of the photon. According to the assumption of Ref. [10], for an optoacoustic transducer with a layered structure, when the thermal and stress confinement conditions of the optoacoustic transducer are met (that is, the width of the pulsed laser must be less than the time for the heat pulse and stress pulse to pass through the light absorption area ($L_{a}=1/\alpha$) in the optoacoustic composite layer [34, 36–38]), the sound pressure (P) of an optoacoustic transducer can be expressed as [10]

(3)P=Kβ∆T,

where β and K are the expansion coefficient and bulk modulus of the medium, respectively.

## 3. Ultrasound Transducer Design and Fabrication

### 3.1. Piezoelectric Transducer

Piezoelectric materials used for ultrasound transducer fabrication contain lead-content materials and lead-free materials. Their properties, including the piezoelectric coefficient ($d_{33}$), dielectric properties, electromechanical coupling coefficient ($k_{t}$), and acoustic impedance, determine the performance of the transducers. In addition, the use of the piezoelectric composite has received extensive attention, because such material has the advantage of enhanced electromechanical coupling, which can help broaden bandwidths and increase energy transfer, resulting in a significant improvement in the signal-to-noise ratio (SNR). Generally, ultrasound transducers have two main types: single-element transducer and array (linear, circular, and 2D array). Compared to the array, the single-element transducer has a relatively low level of complexity in geometry. Meanwhile, the array possesses the capability of dynamic focusing, high frame rate, and real-time measurement [39, 40]. In this section, the latest advancement in piezoelectric materials and the design of transducers is reviewed. Table 1 shows the summary performance of different piezoelectric transducers.

**Table 1:** Performance summary of representative piezoelectric transducers.

Piezoelectric material	Category	$d_{33}$ (pC N^-1^)	$k_{t}$	Center frequency (MHz)	-6 dB BW (%)	Structure	Size (mm×mm)	Reference
PZT-5H	3D printed ceramics	~350	0.55	2.24	35	8×8 2D array	15×15	[41]
PZT-5H	Film	~600	0.4	100-300	23	Single element	—	[42]
Sm-PMN-PT	Ceramic 1-3 composite	1000	0.79	~1	—	16×16 2D array	17.48×17.48	[43]
PMN-PT	Single-crystal 1-3 composite	~1500	0.76	40	82	50 circular array	—	[44]
KNN	Ceramics	237	0.44	18.3	42	64 linear array	3×4.8	[40]
KNN	Single crystal	~490	0.55	82	57.3	Single element	0.4×0.4	[45]
BT	3D printed ceramics	~160	0.47	6.28	41.3	Single element	—	[46]
LiNbO_3_	Single crystal	~40	~0.5	100-300	>40	Single element	0.4×0.4	[47]
NBT	Ceramics	~30	0.35	70.4	52.7	Single element	0.7×0.7	[48]
ZnO	Film	~30	0.28	330	21	Single element	—	[49]
PLLA	Fiber	~10	—	1	—	Single element	5×5	[50]

#### 3.1.1. Lead-Content Piezoelectric Transducers

**Figure 3:**
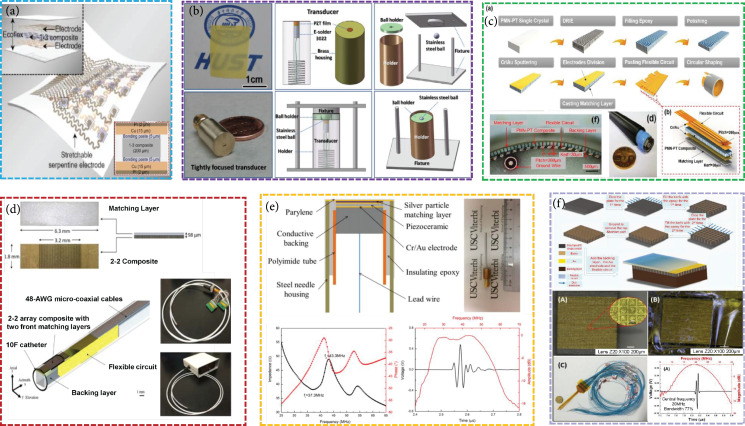
(a) Flexible ultrasound arrays based on the PZT-5H/epoxy 1-3 composites [27], reproduced with permission, copyright 2018, Springer Nature. (b) A 50 MHz ultrasound transducer based on the hydrothermal PZT-5H thick film [57], reproduced with permission, copyright 2016, AIP Publishing. (c) 128-element 0.7PMN-0.3PT 1-3 composite-based circular array [58], reproduced with permission, copyright 2020, IEEE. (d) Miniaturized 64-element side-looking phased array [59], reproduced with permission, copyright 2019, IEEE. (e) High-performance ultrasound needle transducer based on modified PMN-PT ceramic with ultrahigh clamped dielectric permittivity [60], reproduced with permission, copyright 2018, IEEE. (f) 20 MHz 48-element 0.27PIN-0.45PMN-0.28PT single-crystal phased array [61], reproduced with permission, copyright 2020, MDPI.

*(1) Lead Zirconate Titanate (PZT)*. Owing to its higher piezoelectric coefficient ($d_{33}$ ~600 pC N^-1^) and electromechanical coupling coefficient ($k_{t}$ ~0.5), PZT with a perovskite crystal structure has been widely used for fabricating ultrasound transducers [51]. The low mechanical and dielectric losses of PZT benefit various applications in high power ultrasonic therapy [32]. Woo and Roh developed a 3 MHz HIFU transducer based on the PZT-5A ceramic [52]. 3D printing technology has good potential for ultrasound transducer fabrication due to its satisfactory properties, including near-net-shape forming, high green strength, and low binder concentration [41]. Using this technology, Chen et al. fabricated an ~2.24 MHz (-6 dB bandwidth: 35%) single-element transducer based on PZT-5H ceramic [41]. Recently, flexible transducers have been investigated. The Jiang group used the PZT/PDMS 1-3 composite to fabricate a 1~5 MHz flexible ultrasound transducer [53, 54]. Wang et al. designed a ~7.5 MHz flexible ultrasonic transducer based on the PZT-5H 1-3 composite (Figure 3(a)) [27]. Simultaneously, their group described a low-profile membrane-based stretchable ultrasonic transducer [55]. The high-performance PZT-5H 1-3 composites are used to design ~3.5 MHz 10×10 array transducer (-6 dB bandwidth: ~47%, high SNR: ~20.28 dB), multilayered serpentine metal traces as electrical interconnects, and low-modulus elastomer membranes as encapsulation materials [55]. The phased-array transducer can steer and focus the pulse ultrasound beam. Chiu et al. designed a 48-element 20 MHz (-6 dB bandwidth: 42%) phased-array transducer using the PZT-5H ceramic 1-3 composite [56]. With the operational frequency increasing, the thickness of the piezoelectric material is required to be thinner. As the lapping down process of bulk material is difficult and time-consuming, piezoelectric thick films are regarded as a good candidate for high-frequency transducers. Our group reported a PZT thick film simple fabrication technology using a hydrothermal method and obtained a 50 MHz (-6 dB bandwidth: 40%) single-element ultrasound transducer (Figure 3(b)) [57]. In addition, the sol-gel and sol-infiltration technique is successfully used to fabricate a high-frequency ultrasound transducer. Chen et al. built single-element ultrahigh-frequency (100-300 MHz) needle (~mm) ultrasound transducers based on PZT thick films prepared using this technique [42].

*(2) Relaxor-PT*. Lead-content perovskite relaxor-PT single crystals/ceramics, such as lead magnesium niobate-lead titanate (PMN-PT), exhibit significantly high performance in terms of the electromechanical coupling coefficient ($k_{t}$ ~0.94), piezoelectric coefficient ($d_{33}$ ~4100 pC N^-1^), dielectric permittivity (above 13,000), and low dielectric loss (suppressing internal heat generation) [12–18]. The features make them suitable candidates for ultrasound transducers with high sensitivity and broad bandwidth. Using the 0.7PMN-0.3PT single crystal 1-3 composite, Zhang et al. developed a 6.9 MHz (-6 dB bandwidth: 64.6%) 128-element circular array transducer (Figure 3(c)) [58]; Cabrera-Munoz et al. designed a miniaturized 15 MHz (-6 dB bandwidth: 52.2%) 64-element side-looking phased-array catheter (diameter: 3.3 mm) (Figure 3(d)) [59]. Subsequently, Cabrera-Munoz et al. fabricated a 39 MHz (-6 dB bandwidth: 80%) 0.4 mm×0.4 mm needle-type transducer using modified PMN-PT ceramic with ultrahigh clamped permittivity (Figure 3(e)) [60]. Li et al. fabricated a ~40 MHz (-6 dB bandwidth: 82%) 50-element circular array needle-type 1-3 composite transducer (diameter: 1.7 mm) using 0.67PMN-0.33PT single crystal [44]. In recent years, PIN-PMN-PT single crystal has attracted much attention, because it can solve the drawback of relatively low $T_{c}$ of PMN-PT single crystal [61]. Our group prepared a 20 MHz (-6 dB bandwidth: 77%) 48-element phased array based on the 0.27PIN-0.45PMN-0.28PT single crystal 1-3 composite (Figure 3(f)) [61]. In order to further enhance the piezoelectric performance of PMN-PT, the Sm doped PMN-PT material has been successfully developed. Based on Sm-PMN-PT ceramic, Zhang et al. designed the 1 MHz 256-element 2D array [43].

#### 3.1.2. Lead-Free Piezoelectric Transducer

**Figure 4:**
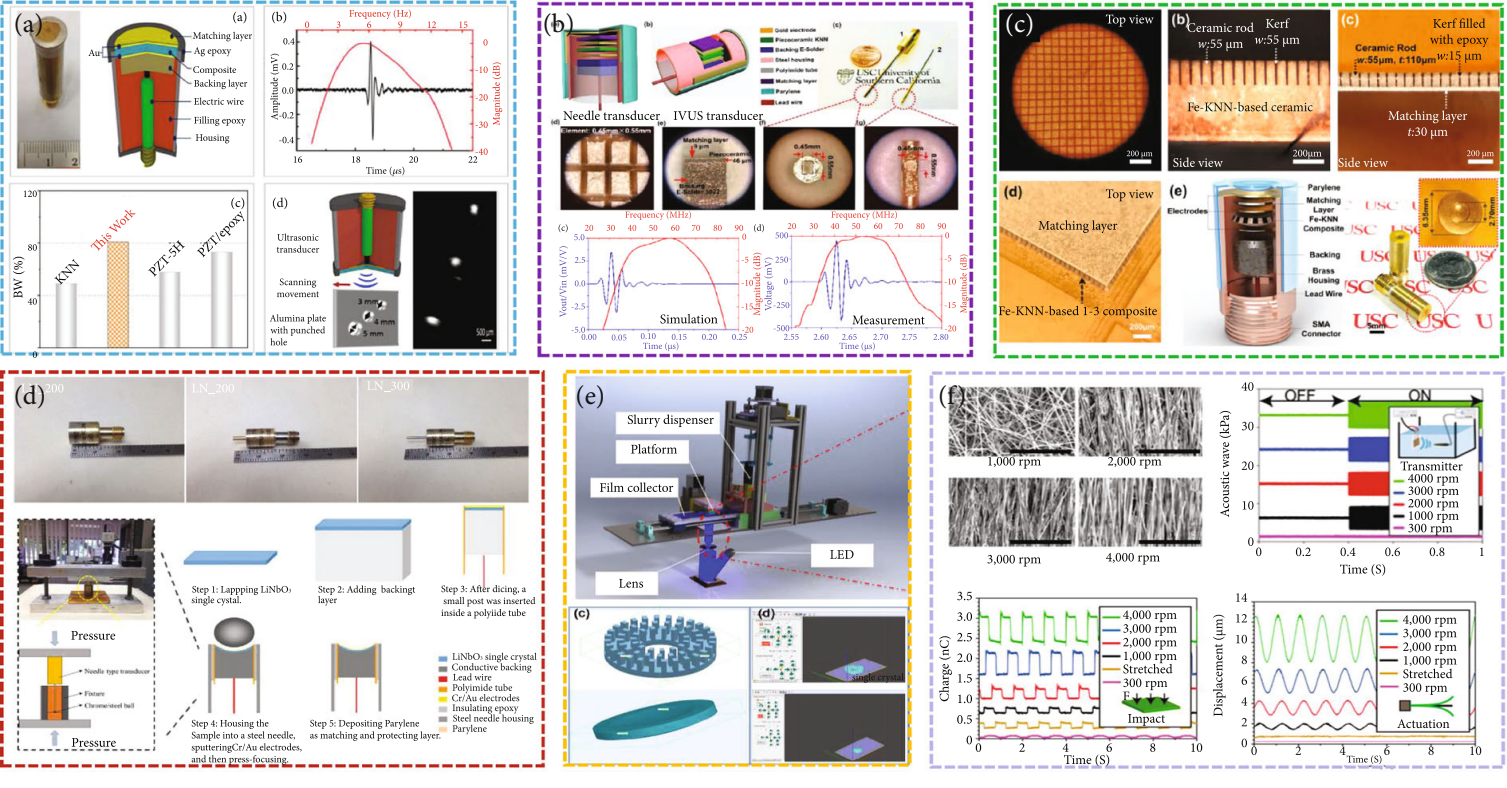
(a) KNN/epoxy 1-3 composite single-element ultrasonic transducer [63], reproduced with permission, copyright 2020, Elsevier. (b) KNN-based ceramic highly sensitive needle transducer [51], reproduced with permission, copyright 2019, IEEE. (c) KNN-based 1-3 composite high-sensitivity ultrasound transducers [64], reproduced with permission, copyright 2019, AIP Publishing. (d) 100-300 MHz LiNbO_3_ ultrasound transducers [47], reproduced with permission, copyright 2016, Springer Nature. (e) 3D printed BaTiO_3_ ultrasound transducer [46], reproduced with permission, copyright 2016, Elsevier. (f) PLLA nanofiber ultrasound transducers [50], reproduced with permission, copyright 2020, Proceedings of the National Academy of Sciences.

*(1) (K,Na)NbO_3_ (KNN)*. The KNN family is the most promising candidate for lead-free piezoelectric ultrasound transducer applications because of its stable piezoelectric properties (~700 pC N^-1^), high $k_{t}$ (~0.6), and relatively high Curie temperature (~302°C). Therefore, KNN-based piezoelectric materials have been extensively investigated for the development of ultrasound transducers [17, 40, 62]. Zheng et al. further improved the piezoelectricity and electromechanical coupling factors and in situ temperature stability of KNN-based ceramics by phase structural engineering [63]. They built a 5 MHz (-6 dB bandwidth: 81%) millimeter transducer based on high-performance KNN 1-3 composites (Figure 4(a)). So far, only few studies have been conducted on array ultrasound transducers with lead-free piezoelectric materials. Zhang et al. designed an 18.3 MHz (-6 dB bandwidth: 42%) 64-element linear array based on KNN ceramics, which has a 75 *μ*m pitch (<λ in water) and is interconnected via a custom flexible circuit [40]. Chen et al. fabricated a 52.6 MHz (-6 dB bandwidth: 64.4%) needle transducer (Figure 4(b)), whose high sensitivity is attributed to the internal microstructure (i.e., phase structure and nanodomains) of KNN ceramics [51]. Subsequently, they developed a high sensitivity, broad -6 dB bandwidth (83%) 16 MHz ultrasound transducer based on KNN 1-3 composites (Figure 4(c)) [64]. Quan et al. fabricated BaZrO_3_ and (Bi_0.5_Na_0.5_) TiO_3_-modified KNN-based textured ceramicsusing the reactive template grain growth method and built an 81 MHz (-6 dB bandwidth: 52%) needle transducer [65]. Compared with KNN ceramics, KNN single crystals have better piezoelectric properties owing to their higher degree of orientation and grain-boundary free microstructures [66]. Recently, a large-sized KNN single crystal with a large $d_{33}$ (670 pC N^-1^) has been achieved by a seed-free solid state crystal growth method. Using the synthesized KNN-based single crystal, our group built a 38 MHz (-6 dB bandwidth: 56%) needle transducer (diameter: 1 mm). Subsequently, our group fabricated a central frequency of an 82 MHz (-6 dB bandwidth: 57.3%) side-looking needle transducer based on a KNN-based single-crystal thick film [45].

*(2) LiNbO_3_*. Among lead-free materials, LiNbO_3_ single crystal has some excellent material properties ($d_{33}$ ~40 pC N^-1^, $k_{t}$ ~0.6, $T_{c}$: ~1210°C) for its use as a transducer material. The Zhang group designed a 75 MHz (-6 dB bandwidth: 92%) single-element transducer (diameter: 6 mm) by using press-focused LiNbO_3_ single crystal [67]. Furthermore, their group fabricated an ultrahigh-frequency (100-300 MHz) single-element needle transducer (Figure 4(d)) [47]. Recently, multilayer polymer-metal structures for acoustic impedance matching have been investigated to avoid reliance on the specific impedance of the materials. Such multilayer structures entail using polymers and metals with different impedances and achieve a specific matching effect by adjusting the thickness of each layer. Yang et al. found that the matching effect of a triple-layer polymer-metal polymer can significantly improve the high-frequency (~90 MHz) broad bandwidth (86.6%) and sensitivity of the ultrasound transducer based on LiNbO_3_ single crystal [68]. Zhang et al. designed a 100 MHz 10-element array of buffer-layer structure, and the ultrasound beam in the azimuth plane in water could be electronically focused to obtain a spatial resolution of 86 *μ*m at the focal depth [69].

*(3) BaTiO_3_ (BT)*. A BT-based ceramic system has various advantages, such as stable electrical properties, good electromechanical coupling (~0.47), and low dielectric loss, but its low $d_{33}$ (~160 pC N^-1^) limits application. BT-based ceramics with improved piezoelectric properties have been achieved by novel structures or by optimizing preparation procedures [70]. Chen et al. fabricated a 6.28 MHz (-6 dB bandwidth: 41.3%) ultrasound transducer based on BT ceramics using 3D printing technology (Figure 4(e)) [46].

*(4) (Bi, Na)TiO_3_ (NBT)*. Although NBT ceramic has strong ferroelectric properties, low dielectric loss, and relatively high Curie temperature ($T_{c}$ ~320°C), its piezoelectric coefficient ($d_{33}$ ~16 pC N^-1^) is low [48]. Fei et al. fabricated Co-doped NBT (NBT-Co) piezoelectric ceramics to improve the piezoelectric properties of NBT ceramics and designed a 0.7×0.7 mm needle ultrasound transducer with a center frequency of 72.7 MHz (-6 dB bandwidth: 52.7%) and f-number close to 1 [48]. Liu et al. fabricated a 2×16 array 82.84 MHz (-6 dB bandwidth: 46.77%) millimeter transducer using an NBT-based thick film [71].

*(5) ZnO*. Traditionally, ZnO ($d_{33}$ ~30 pC N^-1^, $k_{t}$ ~0.28) has been used for the ultrahigh-frequency transducer design because of its low relative dielectric constant (~4) and deposition in quite thin layers (~1 *μ*m) with excellent uniformity on a substrate [49]. Recently, Fei et al. fabricated a 330 MHz (-6 dB bandwidth: 21%) transducer using ZnO film [49].

*(6) Organic Piezoelectric Material*. Organic piezoelectric materials, such as polyvinylidene difluoride (PVDF) ($d_{33}$ ~20-28 pC N^-1^, $k_{t}$ ~0.16) and biopiezoelectric materials, have attracted significant research interest in recent years owing to their low density, high flexibility, and low acoustic impedance [72]. PVDF has low acoustic impedance that is better matched to tissues and has outstanding broadband receiving performance, even with a small sensing area. Therefore, PVDF is mainly used in acoustic detectors [73]. Recently, Curry et al. developed a biodegradable and biocompatible piezoelectric poly(L-lactic acid) (PLLA) nanofiber ($d_{33}$ ~10 pC N^-1^) and fabricated a 1 MHz ultrasound transducer generating an acoustic pressure of 0.3 MPa (Figure 4(f)) [50].

### 3.2. Optoacoustic Transducer

The thermal expansion and light absorption materials are the main components of the optoacoustic transducer. Generally, an ideal thermal expansion material for the optoacoustic transducer is the polydimethylsiloxane (PDMS) because of its high thermal expansion coefficient, low specific heat capacity, and high transparency. Light absorption materials include metal films, carbon nanomaterials, and perovskite. This section introduces research on metal-PDMS composite, carbon-PDMS composite, and perovskite-PDMS composite optoacoustic transducers. Table 2 shows the summary performance of different optoacoustic transducers.

**Table 2:** Performance summary of representative optoacoustic transducers.

Light absorption materials	Transducer’s aperture size (mm)	d (*μ*m)	CF (MHz)	-6 dB BW (%)	η(×10^-2^)	P (MPa)	h (mm)	Pulse laser parameters		Reference
								E(mJ/pulse)	τ (ns)	
Au	—	0.02	~80	~180	—	1.5	0	$1\times 10^{-4}$	5	[74]
CSNP	~10	6	10	210	0.441	+4.8/-1	4.2	3.57	6	[75]
CNTs-xylene	0.22	~1	28.5	~140	—	12.2	3	0.01	2	[76]
CNTs-yarn	10	~5	11.8	179	2.74	+33.6/-10	5	45	5	[6]
CNT array	5	18	20.2	152	0.251	+8.8	2	10	5	[77]
Functionalized CNTs	2.8	38	~100	171	—	1.69	0	—	0.03	[35]
MAPbI_3_	5	0.34	29.2	140	2.97	+15/-10	2	3	5	[10]

Note: d: transducer thickness; CF: center frequency; BW: bandwidth; η: optoacoustic energy conversion efficiency; P: optoacoustic pressure; h: the distance of hydrophone and transducer; E,τ: the energy and width of pulse laser.

#### 3.2.1. Metal-PDMS Composite

Metal-PDMS-based optoacoustic transducer, using gold (Au) [74, 78], chromium (Cr) [79, 80], or germanium (Ge) [81], has already been extensively studied. It can generate high-frequency (~80 MHz) ultrasound with broad bandwidth (-6 dB bandwidth: ~180%) [78–81]. Owing to the low light absorption and high light reflectivity of the metal film, the optoacoustic transducer has low sound pressure [78–82]. Additionally, the metal-PDMS optoacoustic transducer has a low laser damage threshold [78–82]. These reasons limit its biomedical applications.

#### 3.2.2. Carbon Nanomaterials-PDMS Composite

With in-depth research, carbon nanomaterials gradually have become the focus of researchers’ attention because of their excellent optical (broadband light absorption) and thermal (high photothermal conversion efficiency, excellent thermal conductivity, low specific heat capacity, and good thermal diffusivity) properties, such as carbon nanotubes (CNTs), carbon black (CB) [83], graphene [84], carbon nanofibers (CNFs) [85], graphite [86], and candle-soot carbon nanoparticles (CSNPs) [87]. Using carbon nanomaterial-PDMS composites, the optoacoustic transducer has higher optoacoustic conversion efficiency and laser damage threshold than the metal-PDMS optoacoustic transducer.

**Figure 5:**
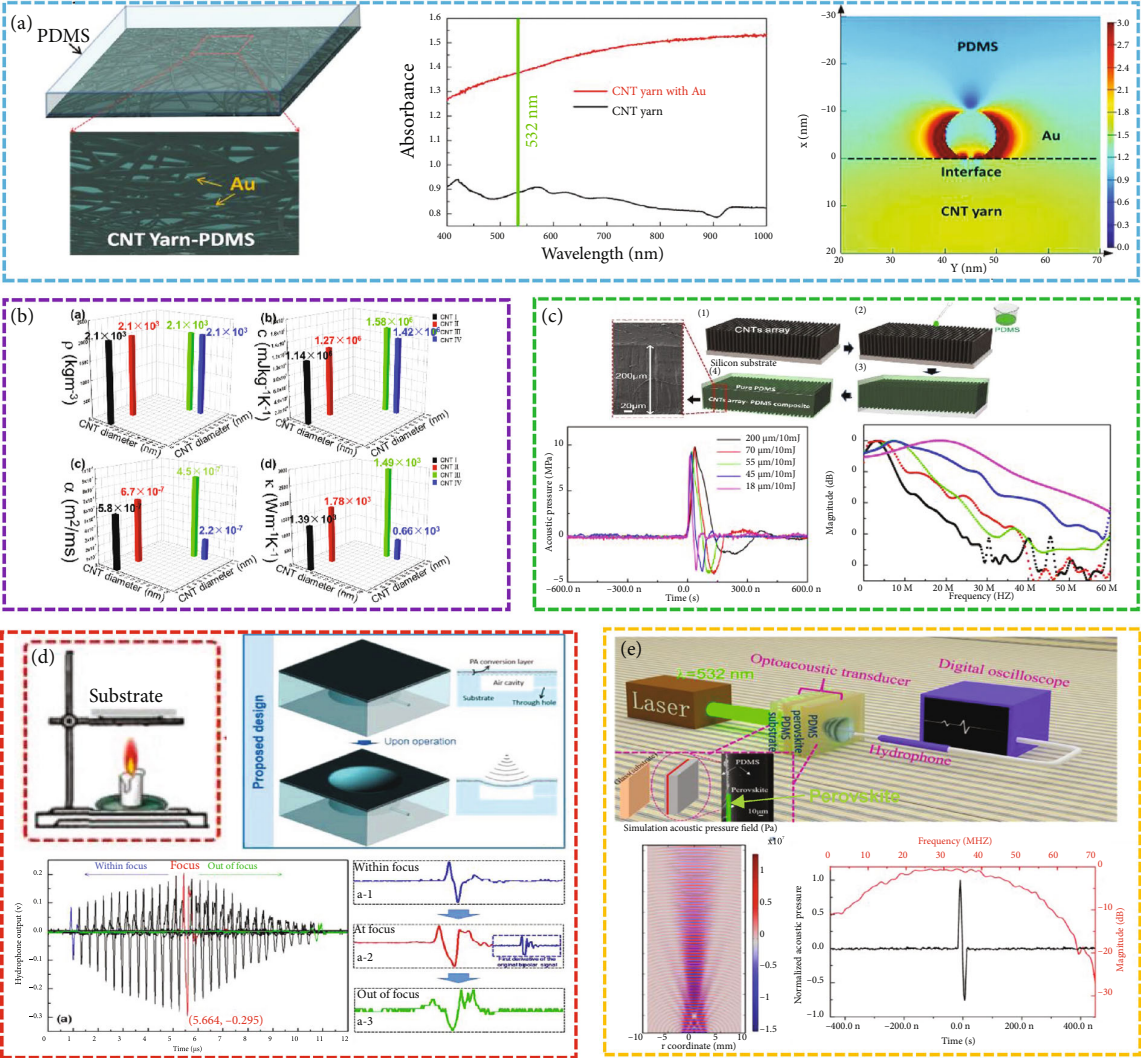
(a) The multilayered CNTs-yarn/Au nanoparticles-PDMS optoacoustic transducer with high energy conversion [6], reproduced with permission, copyright 2018, Elsevier. (b) Effects of CNT thermal conductivity on optoacoustic conversion efficiency [89], reproduced with permission, copyright 2019, Elsevier. (c) The CNT array-PDMS composite optoacoustic transducer [77], reproduced with permission, copyright 2020, Elsevier. (d) The real-time dynamic acoustic focusing of CSNP-PDMS composite optoacoustic transducer [90], reproduced with permission, copyright 2021, Elsevier. (e) The perovskite-PDMS composite optoacoustic transducers [10], reproduced with permission, copyright 2021, Springer Nature.

*(1) CNT-PDMS Composite*. CNTs exhibit a series of unusual properties due to their unique structure and size, making them ideal light absorption materials for use in optoacoustic transducers. Baac et al. demonstrated a gold hybrid CNTs/PDMS optoacoustic transducer by depositing a thin layer of gold on the grown CNT film [88]. They have confirmed that the damage threshold of the CNT-PDMS composite film is 10 times higher than that of the Cr film. In addition, the composite film can withstand extremely high laser energy (>400 mJ cm^-2^) without physical damage and generate strong sound pressure. Our group fabricated high energy conversion ($2.74\times 10^{-2}$) of the optoacoustic transducer using the multilayered CNTs-yarn/Au nanoparticles-PDMS composite, in which Au nanoparticles enhance light absorption through local surface plasma effect (Figure 5(a)) [6]. Subsequently, our group indicated that the great thermal conductivity of CNTs can generate a stronger optoacoustic signal in the 1-3 composite structure (Figure 5(b)) [89]. Then, our group built an optoacoustic transducer based on the CNT array-PDMS composite with anisotropic thermal conductivity. The frequency modulation (~20 MHz) can be achieved by controlling this composite’s thickness (Figure 5(c)). Our results have proved that the working frequency of the transducer is inversely proportional to the thickness of the CNT array-PDMS composite [77]. Furthermore, our group fabricated a self-healing optoacoustic transducer based on CNT polymer. Even after 10 times of damage and healing, the output sound pressure still maintains at 92.3% of the original value [25]. Silva et al. demonstrated an optoacoustic transducer that generated ~80 MHz (-6 dB bandwidth: 170%) ultrasound pulses with functionalized CNTs and 30 ps pulse laser [35]. The functionalized CNTs promote ultrafast dissipation of heat to the PDMS. The picosecond pulse laser can improve the bandwidth of the optoacoustic transducer.

*(2) CSNP-PDMS Composite*. CSNPs can be produced during the burning process of candles and have demonstrated the advantages of a spherical shape (large surface-to-volume ratio), low specific heat capacity (~750 J kg^-1^ K^-1^), low cost, and fabrication simplicity for developing highly efficient optoacoustic transmitters [10, 75]. Under consistent experimental conditions, the optoacoustic conversion efficiency of the CSNP-PDMS composites was higher than that of the CB- or CNF-PDMS composites [37]. Using CSNP-PDMS composites, our group and collaborators designed a novel optoacoustic transmitter including the transducer and air cavity, which achieved real-time dynamic acoustic focus by adjusting the air pressure of the cavity (Figure 5(d)). Furthermore, the negative optoacoustic pressure was significantly improved, which could provide additional advantages for ultrasonic cavitation applications [90].

#### 3.2.3. Lead Halide Perovskite-PDMS Composites

Taking advantage of low specific heat capacity (~308 J kg^-1^ K^-1^), low thermal conductivity (~0.5 W m^-1^ K^-1^), small thermal diffusion coefficient (0.145 mm^2^ s^−1^), and high absorption coefficient (6.7 *μ*m^-1^) of perovskites, our group designed a lead halide perovskite-PDMS-stack structure optoacoustic transducer. The theoretically calculated phonon spectrum shows that the overlap of optical phonons and acoustic phonons leads to the upconversion of acoustic phonons and thus results in a small thermal diffusion coefficient of methylamine lead iodine perovskite. The small thermal diffusion coefficient can generate a strong thermal localization effect on the perovskite layer, which enhances the energy conversion efficiency of the optoacoustic transducer. Figure 5(e) shows that the central frequency and -6 dB bandwidth of the ultrasound wave are at 29.2 MHz and 40.8 MHz, respectively. When the laser energy was 3 mJ/pulse, the peak-to-peak value of the acoustic pressure was 24.89 MPa, and the optoacoustic conversion efficiency was $2.97\times 10^{-2}$ [10].

## 4. Ultrasound Transducers for Biomedical Applications

### 4.1. Piezoelectric Transducer for Biomedical Applications

#### 4.1.1. Medical Imaging

**Figure 6:**
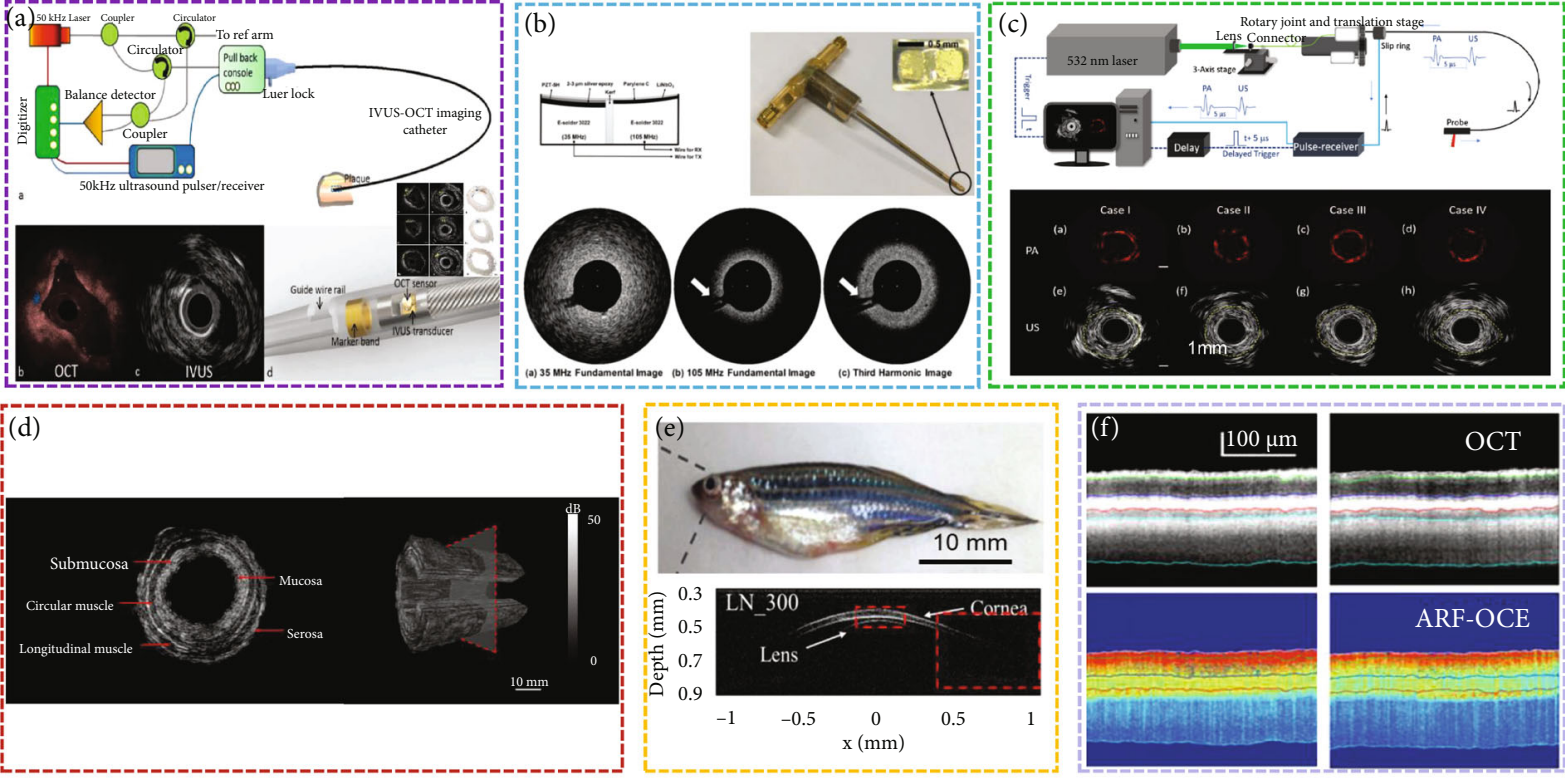
Piezoelectric ultrasound imaging. (a) Ultrafast IVUS-OCT system and miniaturized catheter for atherosclerotic plaque imaging [92], reproduced with permission, copyright 2015, Springer Nature. (b) 35 MHz/105 MHz dual-element focused transducer for IVUS imaging [22], reproduced with permission, copyright 2018, MDPI. (c) Dual-modality photoacoustic and ultrasound endoscopy imaging [97], reproduced with permission, copyright 2019, Elsevier. (d) 128-element 6.8 MHz circular array for the 2D and 3D endoscopic image of the swine intestine [58], reproduced with permission, copyright 2020, IEEE. (e) Ultrahigh-frequency (~300 MHz) ultrasonic transducers for biomicroscopy imaging of zebrafish eyes [47], reproduced with permission, copyright 2016, Springer Nature. (f) ARF-OCE mapping for the retinal layers [98], reproduced with permission, copyright 2018, Association for Research in Vision and Ophthalmology.

*(1) Intravascular Ultrasound (IVUS) Imaging*. IVUS imaging is an important tool in the diagnosis of cardiovascular diseases and has been widely used for clinical diagnosis. Owing to its capability of directly imaging the vessel wall, IVUS can provide an accurate evaluation of lumen size, plaque characteristics, and calcium content [91, 92]. At present, dual-frequency and multimodality technologies are combined with IVUS imaging (Figures 6(a)–6(c)).

*(1)1. Superharmonic Imaging*. Suitable imaging depth and higher resolution imaging (<100 *μ*m) can be achieved by using a dual-frequency approach by transmitting at a lower frequency and receiving at much higher frequencies to contrast imaging. Furthermore, researchers have demonstrated the ability to use microbubble superharmonic signals to enhance the resolution of functional imaging [22]. Dual-frequency transducers capable of detecting microbubble superharmonics have shown promise as a new contrast-enhanced IVUS platform [93]. Lee et al. developed a 35 MHz transducer to transmit ultrasound and a 105 MHz transducer for the reception of third harmonic for intravascular ultrasound tissue imaging (axial resolution: 25 *μ*m and lateral resolution: 46 *μ*m) (Figure 6(b)) [22]. The third harmonic imaging acquired by the developed transducer is capable of providing a deeper imaging depth (1.4 mm) than the 105 MHz fundamental imaging. Dual-frequency IVUS transducers require each element of the transducer to be connected by a coaxial cable. Consequently, at least two coaxial cables should be used for a catheter to increase the diameter of the catheter, which may hinder the clinical application. Su et al. proposed a catheter consisting of a dual-frequency transducer for intravascular ultrasound. Both ultrasonic elements with different frequencies were connected to one coaxial cable to simplify the connection [94].

*(1)2. Acoustic Radiation Force (ARF) Elasticity Imaging*. The challenge in predicting plaque ruptures depends on precise knowledge of the mechanical properties of the arterial wall and the plaque. Owing to the minimal contrast between different types of soft tissues, the sensitivity and specificity of IVUS for detecting the composition of a plaque are poor. Thus, ARF elasticity imaging has been developed by researchers as an alternative to conventional ultrasound elastography. It can be used to distinguish various components of plaques and assess the mechanical properties of arterial walls. Shih et al. designed a dual-frequency IVUS transducer for elasticity imaging, where the propagation of shear waves was induced by an 8.5 MHz ultrasound transducer, and the 31 MHz imaging transducer monitored the elastic properties of plaques and vessels [95]. The stiffness distributions of the atherosclerotic aorta from a rabbit and shear wave velocity of the lipid-rich plaques and arterial walls were 0.38±0.19 m s^-1^ and 3.45±0.45 m s^-1^, respectively.

*(1)3. Dual-Modality Photoacoustic and Ultrasound (PAUS) Imaging*. In IVUS, the contrast between the lipid-rich region and other soft tissues is confined. Intravascular photoacoustic (IVPA) imaging seems to be an alternative method to address this issue to detect the lipid pool and atherosclerotic calcification. In this hybrid imaging process, a tiny single-element ultrasound transducer integrated with a laser fiber and driven by a rotational shaft is placed at an appropriate position inside the blood vessel to detect the photoacoustic signals inside the tissue. As we all know, analyses of the photoacoustic spectrum have already been proven as an effective method to expose significant differences between malignant and normal tissue regions [96]. Consequently, frequency domain analyses seem to be a beneficial supplement in IVPA technology and could be potentially used for the characterization of atherosclerotic plaques [66]. In addition, Li et al. designed a dual-modality PAUS system for endoscopic imaging [97], which showed enhanced bandwidths of the ultrasound transducer and improved SNR of PAUS images (Figure 6(c)).

*(2) Internal Organ Endoscopic Ultrasound Imaging (EUS)*. EUS is a diagnostic imaging method that uses ultrasound to obtain images of internal organs in the human body, such as the chest, abdomen, and colon. It can be used to visualize the organ wall and surrounding structures. Zhang et al. developed a 6.8 MHz 128-element endoscopic ultrasound array to obtain 3D imaging of a healthy swine intestine (Figure 6(d)) [58]. A newly emerging endoscopic technique, called photoacoustic endoscopy (PAE), could be an important complementing procedure in diagnosing GI tract diseases because it is well suited to provide high-resolution microvasculature imaging with rich spectral and functional information of the tissue. Yang et al. described a 3.8 mm diameter side-scanning PAE-EUS probe, which realizes simultaneous PAUS imaging of internal organs [99]. Wireless capsule endoscopy enables remote diagnostic assessment of the gastrointestinal tract in a painless procedure. Wang et al. presented a mechanical scanning device incorporating a 39 MHz high-frequency transducer, which can offer good image resolution (∼60 *μ*m) for the lumen wall of the porcine small intestine [100].

*(3) Ophthalmic Ultrasound Imaging*. Ultrasound biomicroscopy is used for real-time diagnosis of anterior segmental diseases with a relatively deep imaging and a large field of view, regardless of whether the suspicious lesion is in optically transparent or opaque media [51, 101]. Fei et al. used the ~300 MHz single-element ultrasound transducer to obtain the ultrasonic biomicroscopy image of zebrafish eyes, whose resolution is up to ~10 *μ*m, comparable to the resolution of OCT (Figure 6(e)) [47]. The elastic properties of the cornea are crucial for human vision. Therefore, measuring the elasticity distribution of the cornea is important for evaluating corneal pathologies and the efficacy of corneal treatment, particularly during the early stages of corneal sclerosis. Qu et al. used ARF optical coherence elastography (ARF-OCE) to map out the elasticity of retinal layers in healthy and diseased in vivo rabbit models (Figure 6(f)) [98].

#### 4.1.2. Acoustic Tweezers

Optical force, acoustic force, and magnetic force and electrophoresis can be used to manipulate single particles, cells, and organisms for many applications in biology, chemistry, engineering, and physics. Compared with optical, electrical, and magnetic counterparts, acoustic tweezers are relatively noninvasive to biological objects and applicable to most microparticles [102, 103]. Acoustic waves are capable of exerting acoustic radiation forces to levitate particles with a wide range of sizes and materials through air and water. As a promising microparticle manipulator, single beam acoustic tweezers (SBATs) are attractive [57]. They have the advantage of providing a significant trapping force and offering deep penetration, showing great potential for in vivo and clinical applications.

**Figure 7:**
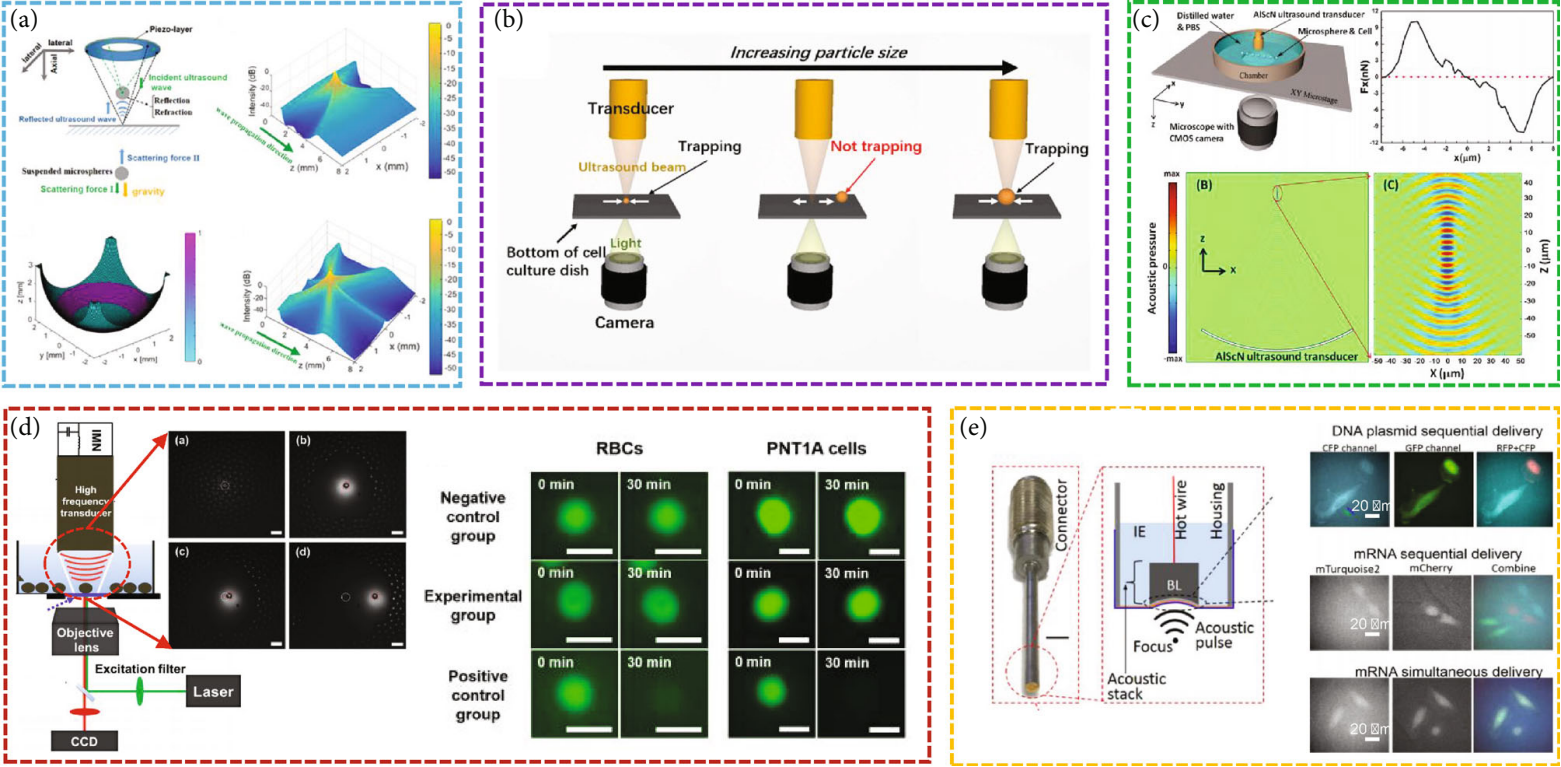
(a) 60 MHz focused ring ultrasonic transducer for SBAT [107], reproduced with permission, copyright 2019, AIP Publishing. (b) SBAT manipulator for microspheres or cells of certain selected sizes [26], reproduced with permission, copyright 2021, Elsevier. (c) ∼230 MHz self-focused ultrasound transducer for individual cell manipulation [110], reproduced with permission, copyright 2017, American Chemical Society. (d) 526 MHz single-element ultrasound transducer for manipulating microparticle sized from 3 *μ*m to 100 *μ*m [113], reproduced with permission, copyright 2017, Springer Nature. (e) High-frequency ultrasound tweezer for manipulating single-cell intracellular delivery of DNA plasmids and mRNA into adjacent cells [114], reproduced with permission, copyright 2017, Springer Nature.

Controlling cell functions for research and therapeutic purposes may open new strategies for the treatment of many diseases. Chen et al. demonstrated that the 3D ultrathin piezoelectric element could generate a 40 MHz helical-like ultrasonic field, which can efficiently allow noncontact trapping and manipulation of suspended microparticles and biological cells [104]. Lim et al. presented the manipulation of a single cell or multiple cells using a 30 MHz linear array transducer [105]. Lim et al. proposed an automatic and reliable cancer cell classification method based on a 50 MHz SBAT, which can obtain a series of images of deformed human breast cancer cells [106]. Our group built a 50 MHz f-number of 0.9 ultrasound transducer, which can manipulate a 10 *μ*m microparticle in distilled water [57]. Chen et al. built a 60 MHz SBAT based on a focused ring ultrasonic transducer, which can levitate and manipulate microparticles (Figure 7(a)) [107]. Lam et al. demonstrated that a 60 MHz SBAT could manipulate an individual red blood cell and a single 1.6 mm diameter fertilized zebrafish egg, respectively [108]. Lee et al. employed an 86 MHz ultrasound transducer to manipulate and identify live single cells [109]. Li et al. reported the selective trapping of microspheres by acoustic force using a >100 MHz SBAT (Figure 7(b)) [26]. They found that setting the SBAT shape and wavelength can manipulate microspheres or cells of certain selected sizes. Our group fabricated an ~230 MHz self-focused SBAT ultrasound transducer, which has a narrow lateral beam width (~8.2 *μ*m). The developed SBAT can continuously manipulate individual 10 *μ*m epidermoid carcinoma cells (Figure 7(c)) [110]. Fei et al. fabricated a needle ultrasonic transducer with a center frequency higher than 300 MHz (-6 dB bandwidth: >64%). The focused acoustic microbeam produced by the transducer can manipulate individual 3 *μ*m microspheres [111]. Chen et al. studied adjustable multiscale SBAT based on a 526 MHz single-element ultrasonic transducer that can flexibly change the size of the “tweezers” in the same manner as ordinary metal tweezers [112].

The mechanical properties of cells play a key role in various cellular functions, such as proliferation, migration, and gene expression. SBATs are a promising technology for the quantification of the mechanical performance of cells. For this purpose, Kim et al. developed a tightly focused 153 MHz ultrasound transducer to grab and separate a single cell from a heterogeneous cell sample and to analyze the physical and functional characteristics of cells (Figure 7(d)) [113]. Hwang et al. demonstrated that a single-beam acoustic trapping technique can be utilized to examine the mechanical properties of breast cancer cells [115]. In addition, the role of cell mechanics in cancer cells has resulted in the identification of new mechanisms of therapy resistance. Their group demonstrated that 30 MHz SBAT can be applied to quantify the deformability of adherent breast cancer cell lines [116] and to explore the elastic properties of pre-B acute lymphocytic leukemia cells [117]. Hwang et al. developed 193 MHz acoustic tweezers to trap 5 *μ*m fibronectin- (FNT-) coated microbead and demonstrated its potential to study intracellular calcium signaling by FNT binding to human breast cancer cells [118].

There have been limited transfection techniques that can deliver multiple types of active molecules simultaneously into single cells with high precision and low cytotoxicity. Yoon et al. introduced a new transfection technique that utilizes the center frequency over 150 MHz ultrasound without any contrast agents inducing intracellular delivery of exogenous molecules [119]. Recently, they reported that a >150 MHz high-frequency ultrasound-based remote intracellular delivery technique capable of delivering multiple DNA plasmids, messenger RNAs, and recombinant proteins has been developed. This technique allows high spatiotemporal visualization and analysis of gene and protein expressions, as well as single-cell gene editing (acoustic transfection). They found that ultrahigh-frequency ultrasound can directly deliver genes and proteins into the cytoplasm without microbubbles (Figure 7(e)) [114].

#### 4.1.3. Other Medical Applications

Recently, researchers have developed implanted piezoelectric transducers to disrupt the blood-brain barrier and facilitate the delivery of drugs into the brain [50, 120]. In addition, a 1.1 MHz ultrasound transducer was applied to modulate the cholinergic anti-inflammatory pathway and to reduce cytokine response to endotoxin to the same levels as implant-based vagus nerve stimulation [121]. In order to provide remarkable insights into the cardiovascular disease diagnosis and prognosis, Wang et al. fabricated a 7.5 MHz flexible ultrasound transducer to capture blood pressure waveforms at deeply embedded arterial and venous sites [27].

### 4.2. Optoacoustic Transducer for Medical Application

#### 4.2.1. All-Optical Ultrasound Imaging

**Figure 8:**
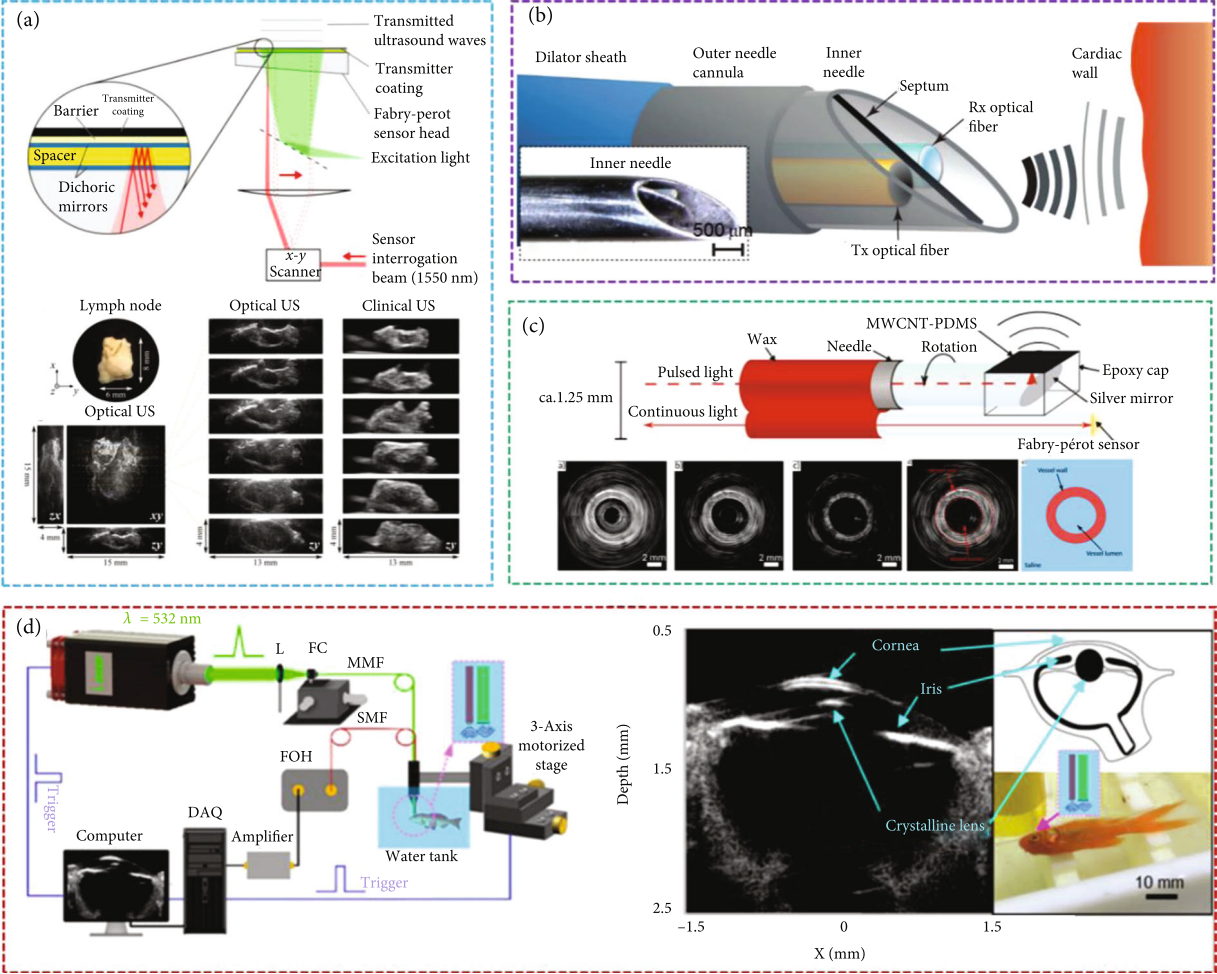
(a) All-optical ultrasound imaging of ex vivo human lymph node [122], reproduced with permission, copyright 2021, IEEE. (b) All-optical fiber ultrasound system for heart motion detection [123], reproduced with permission, copyright 2017, Springer Nature. (c) All-optical ultrasound technology for IVUS imaging [124], reproduced with permission, copyright 2019, Springer Nature. (d) All-optical ultrasound imaging system for fish eye imaging [10], reproduced with permission, copyright 2021, Springer Nature.

The optoacoustic transducer can generate ultrasound pulses with MPa-level peak pressures, offering the potential for improved image quality and higher resolution for tissue imaging. Pham et al. presented a broadband all-optical plane-wave ultrasound imaging system for high-resolution and high-fidelity 3D ultrasound images of ex vivo human lymph nodes (Figure 8(a)) [122]. Finlay et al. built an optoacoustic transducer with a center frequency of ~20 MHz (-6 dB bandwidth: 132.5%), which was integrated within a custom inner transseptal needle to obtain all-optical ultrasound imaging of multiple locations within a swine heart (Figure 8(b)) [123]. Colchester et al. designed an optoacoustic transducer with a center frequency of ~20 MHz (-6 dB bandwidth: 156.5%) [124]. They built an IVUS system using all-optical ultrasound technology to realize in vitro imaging of porcine aorta sections (Figure 8(c)). Noimark et al. fabricated a 28.5 MHz optoacoustic transducer (-6 dB bandwidths: ~140%) at the end of 200 *μ*m optical fiber, which can achieve high-resolution all-optical ultrasound imaging of the porcine aorta in vitro [76]. In 2021, our group developed an all-optical ultrasound imaging system with a 29.2 MHz perovskite-PDMS composite optoacoustic transducer. Using this system, the eye ultrasound imaging of a fish with high resolution and high SNR can be obtained, where the structures of the cornea, iris, and lens surface are clearly visible (Figure 8(d)) [10].

#### 4.2.2. Optoacoustic Medical Therapy

**Figure 9:**
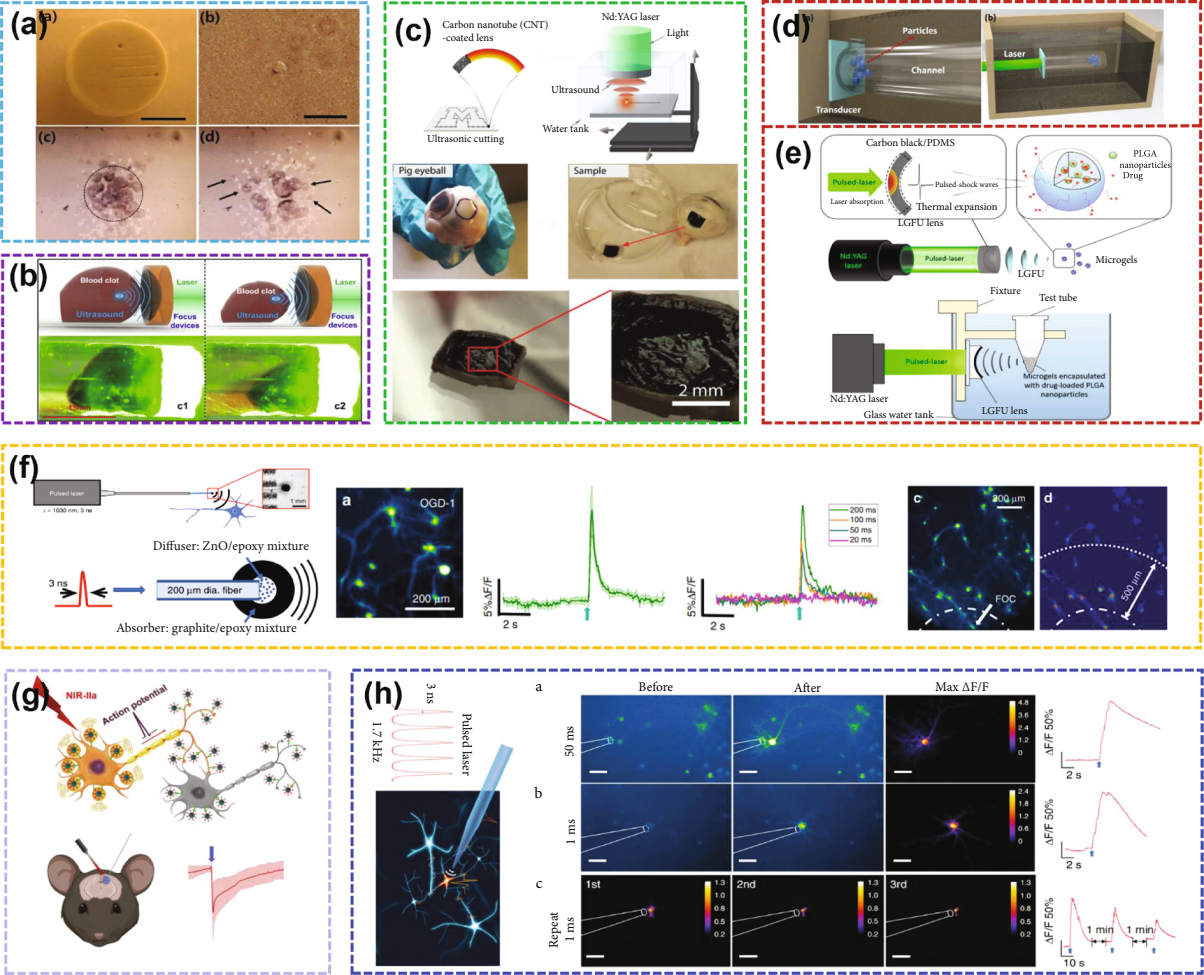
(a) Microscale fragmentation of the solid materials by the LGFU [125], reproduced with permission, copyright 2012, Springer Nature. (b) The optoacoustic transducer for thrombolysis [25], reproduced with permission, copyright 2020, Elsevier. (c) The optoacoustic transducer for cutting tissue [126], reproduced with permission, copyright 2017, John Wiley and Sons. (d) The optoacoustic transducer for microparticle movement control [6], reproduced with permission, copyright 2018, Elsevier. (e) The optoacoustic transducer for drug delivery [128], reproduced with permission, copyright 2015, Elsevier. (f) The optoacoustic transducer for neuron stimulation [23], reproduced with permission, copyright 2020, Springer Nature. (g) The optoacoustic nanotransducers for targeted neuromodulation [129], reproduced with permission, copyright 2021, Elsevier. (h) The optoacoustic transducer for stimulating single neurons [130], reproduced with permission, copyright 2021, Springer Nature.

*(1) Ultrasound Operation*. With the application of an optoacoustic lens, coating the optoacoustic composite material on the concave surface to generate focused ultrasound, the sound pressure of an optoacoustic transducer has been greatly improved. Baac et al. used laser-generated focused ultrasound (LGFU) to perform an experiment on an artificial kidney-stone model [125]. They used LGFU to break the polymer coating, demonstrating its potential application in lithotripsy treatment (Figure 9(a)). Subsequently, our group performed an ultrasound thrombolysis experiment using a self-healing optoacoustic transducer (Figure 9(b)) [25]. Lee et al. demonstrated that LGFU can be used for high-precision cavitation cutting that is applicable to more complex shapes [126]. They removed the choroid from the pig’s eye and then applied LGFU to the choroid surface. Two small ablation grooves are observed on the choroid after drying the sample, which implies the achievement of mechanical ablation (Figure 9(c)).

*(2) Drug Delivery*. High-intensity optoacoustic waves can generate acoustic cavitation which is effective for the control of drug release [127]. Furthermore, the low duty cycle of optoacoustic waves can reduce ultrasound-induced heating, thereby avoiding the detrimental effects on surrounding tissues. Our group developed a new type of optoacoustic transducer with a PDMS/Au-CNT yarn-PDMS structure using multilayer CNT yarn and successfully applied it to manipulate particles in a certain direction (Figure 9(d)) [6]. Di et al. used a focused optoacoustic transducer based on carbon-black/PDMS composite to promote drug release. As shown in Figure 9(e), when laser-generated ultrasound excites the microgels, gradual release of the drug from poly(lactic-co-glycolic acid) nanoparticles is promoted due to the cavitation effects at the microgels and oscillation of the microgel shells [128]. These results provide guidelines for further in vivo potential clinical applications.

*(3) Nerve Stimulation*. Laser-generated ultrasound is an emerging modality for neuromodulation [126]. Jiang et al. reported an optoacoustic neural stimulator using a miniaturized fiber-optic converter, which could generate ~1 MHz omnidirectional ultrasound wave (Figure 9(f)) [23]. With studies on living animals, the application of laser-generated ultrasound to neuromodulation and brain stimulation of the human body is expected to be available in the future. Subsequently, their group reported semiconducting polymer nanoparticle-based targeted photoacoustic nanotransducers (PANs) for neural stimulation [129]. PANs can be surface-modified to selectively bind onto neurons; laser-generated ultrasound produced by PANs can modulate neuron activities (Figure 9(g)). Recently, their group also fabricated a tapered fiber optoacoustic emitter to generate an ultrasound field with a high spatial precision of 39.6 *μ*m, which can modulate single neurons or subcellular structures (Figure 9(h)), such as axons and dendrites [130].

## 5. Conclusions and Outlook

**Figure 10:**
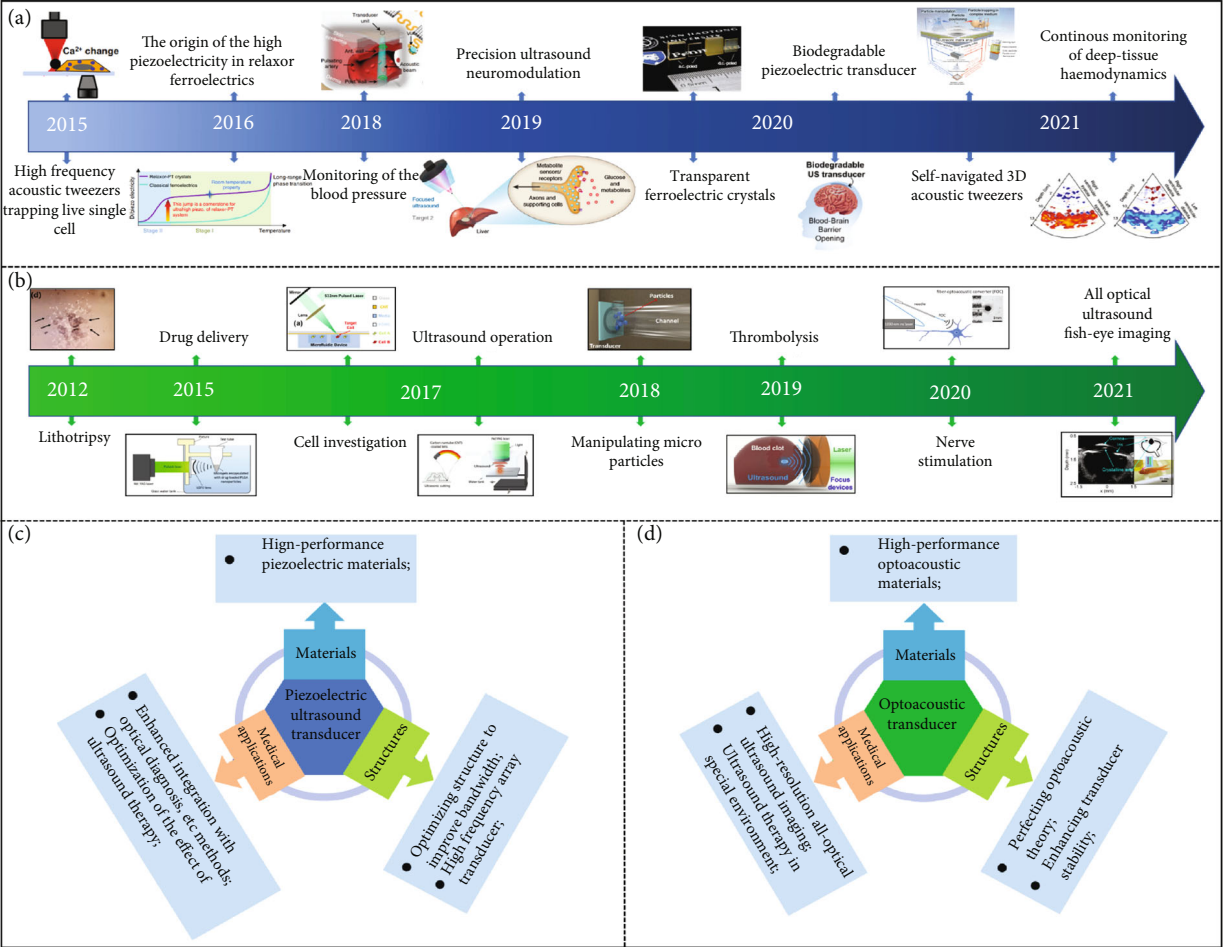
Summary of crucial developments in piezoelectric (a) and optoacoustic (b) [6, 10, 12, 18, 20, 23–25, 27, 103, 118, 120, 121, 125, 126, 128] transducers, reproduced with permission. Research prospects of piezoelectric (c) and optoacoustic (d) transducers.

This review summarizes some recent advances in the field of an ultrasound transducer that has been widely used in clinical diagnosis and treatments, such as biomedical imaging, thrombolysis, cell manipulation, drug delivery, and neuromodulation. Plenty of researchers around the world, including the United States, the United Kingdom, China, and South Korea, are focusing on this field. Figure 10 shows the summary and prospective crucial development for ultrasound transducers recently. As explained in this review, ultrasound transducers could be divided into two major categories: piezoelectric transducers and optoacoustic transducers. A piezoelectric transducer which belongs to traditional ultrasound devices has been extensively investigated for a wide range of biomedical engineering applications and is being revolutionized by advances in microelectronic technologies. The optoacoustic transducer emerges as a promising candidate for biomedical engineering applications, due to its simple preparation processes, antielectromagnetic interference, and broad bandwidth. The progresses of ultrasound transducers from the perspective of material strategies, design considerations, and biomedical engineering applications have been discussed systematically.

Because the material selection and structure design of ultrasound transducers have vital influences on its acoustic performance, seeking new material with excellent properties and optimizing device structure are two eternal themes for ultrasound transducer development. Recent researches on piezoelectric material have made a great breakthrough that Sm-PMN-PT single crystal with giant piezoelectricity and transparent PMN-PT single crystal with ultrahigh piezoelectricity have been invented [15, 16, 18], providing a new strategy for ultrasound transducer fabrication. From the perspective of environmental protection, new nontoxic materials with such super piezoelectricity are in demand for next-generation piezoelectric transducers. Different from piezoelectric devices, the optoacoustic transducer has a more complex energy conversion process. In order to precisely control the performance of the optoacoustic transducer, device physics needs to be further explored. At present, carbon nanomaterial-PDMS composites dominate optoacoustic materials, but the energy conversion efficiency is still low. For next-generation optoacoustic transducer fabrication, novel materials with higher optoacoustic energy conversion coefficients are required. For convenience in biomedical engineering application, the development trend of the ultrasound transducer is towards package miniaturization, array design, and multifunctional integration. Moreover, the continuous influx of new technologies, such as 3D printing, flexible electronics, and artificial intelligent, are expected to bring an innovation concept for transducer design.

Finally, we hope this work can provide a summary of current ultrasound transducer developments for further theoretical study and inspire better structure design of the ultrasound transducer for biomedical engineering applications in the future.
